# Promising Detoxification Strategies to Mitigate Mycotoxins in Food and Feed

**DOI:** 10.3390/toxins10030116

**Published:** 2018-03-09

**Authors:** Yousef I. Hassan, Ting Zhou

**Affiliations:** Guelph Research and Development Centre, Agriculture and Agri-Food Canada, 93 Stone Road West, Guelph, ON N1G 5C9, Canada; Yousef.Hassan@AGR.GC.CA

Mycotoxins are secondary fungal metabolites associated with adverse human health and animal productivity consequences. Annual costs connected with mycotoxin occurrences in food/feed are continuing to rise. It is estimated that close to five billion dollars are lost yearly in association with fungal infections and crop contamination with mycotoxins within the North American region alone. More recent evaluations valued losses associated with aflatoxin (AF) contaminations within the corn industry to reach as high as US$1.68 billion annually in the United States [1]. Similarly, the U.S. swine industry was reported to face current losses (in the form of weight gain reduction) due to fumonisins contamination in dried distillers’ grain and solubles (DDGS) of $9 million annually [2]. This value represents only those losses attributable to one mycotoxin on one adverse outcome in one species. In Europe, deoxynivalenol (DON) is typically found in more than 50% of investigated samples [3]. When 18,884 samples collected between 2007 and 2012 from member-states of the European Union (EU) and Norway were investigated for mycotoxins, DON was found in 44.6%, 43.5%, and 75.2% of unprocessed grains, food, and feed samples, respectively [4]. The same pattern is also encountered in North Asia, with DON being the main contaminant (present in 92% of all tested samples) with average levels of 1154 ppb (part per billion) [5]. The latest multi-city survey conducted in China on the occurrence of DON in different cereal-based products indicated that more than 80% of the analyzed samples were positive with DON levels ranging between 0.1 and 2511.7 μg/kg [6].

After more than five decades of continuous mycotoxin-mitigation research, our understanding started to reach a pinnacle point where biological and enzymatic means can be used to address such toxins. Moreover, advances in food and feed processing techniques (such as cold atmospheric pressure plasma, hot air and infrared rays roasting, neutral electrolyzed water, etc.) coupled with state-of-the-art molecular research tools are leading the way for optimizing empirical and feasible solutions.

In light of the above facts, the focus of this special issue of *Toxins* was to look into the most recent advances related to mitigating mycotoxin contamination in food and feed. Multiple recent microbial and enzymatic investigations are included and many novel and promising techniques for food/feed applications are covered.

Wilson et al. [7] reported the screening of plant and soil samples for microorganisms capable of degrading trichothecenes and eventually identified two mixed cultures consistently decreasing DON levels through oxidation to 3-keto-DON. Another study screened 43 bacterial isolates and identified *Bacillus shackletonii* L7, which is capable of reducing aflatoxin B1 (AFB_1_), AFB_2_, and AFM_1_ [8] where a thermostable-enzyme enriched in the culture’s supernatant was purified with an estimated molecular mass of 22 kDa. Moreover, a separate study focused on elucidating how one bio-control agent, *Sporobolomyces* sp., targets and degrades patulin (PAT), a commonly encountered mycotoxin that contaminates apple and cider products [9]. The involved mechanism behind this microorganism’s ability to degrade PAT was shown to be inducible with a rapid degradation of PAT, especially when the cells of this agent are exposed to low concentrations of PAT ahead of time. Furthermore, the mechanism(s) behind the degradation of PAT by another yeast isolate, *Pichia caribbica*, was examined. The collected results indicated the involvement of an enzymatic mechanism [10], while the rigorous proteomics analysis (with two-dimensional gel electrophoresis) revealed the upregulation of multiple proteins involved in the cellular metabolism and/or stress response which could be responsible for PAT degradation at the same time.

The presented special issue additionally reported on expanding the current empirical utilization of innovative mitigation strategies to control mycotoxins in actual farm settings. The use of neutral electrolyzed water to prevent aflatoxicosis in Turkey poults was among the promising studies that were shared by Gómez-Espinosa et al. [11]. As reported, alterations of serum biochemical constituents, enzyme activities, relative organs weights, and morphological changes associated with AF(s) were all mitigated by using the described neutral electrolyzed water detoxification procedure. Another novel investigation led by Bosch et al. scrutinized the use of cold atmospheric pressure plasma for the degradation of multiple mycotoxins including AAL toxin, enniatin A, enniatin B, fumonisin B_1_, sterigmatocystin, DON, T2-toxin, and zearalenone (ZEA) [12]. The results reflected a significant influence of the involved mycotoxin’s structure in addition to the matrix on the overall degradation rates. The results collectively indicated the suitability of the introduced approach for the decontamination of mycotoxins in food commodities where mycotoxins are confined to or enriched on surfaces such as cereal grains. Roasting with the use of infrared or static hot air was investigated for its ability to decontaminate AFs in hazelnuts [13]. Both traditional static hot-air roasting and infrared rays roasting methods were effective (85–95% reduction) when temperatures of 140 °C for 40 min were maintained, but infrared rays proved to be slightly better in this regard. More importantly, the nutritional quality and lipid profile of all tested hazelnut varieties were not affected after such roasting. Ultraviolet irradiation was also suggested to reduce AF(s) genotoxicity and carcinogenicity [14]. In order to define the final by-products of this non-specific degradation method, especially in edible oils, an Ultra Performance Liquid Chromatograph-Thermo Quadrupole Exactive Focus Mass Spectrometry/Mass Spectrometry (UPLC-TQEF-MS/MS) approach was used. The obtained high-resolution mass spectra reflected two main products while the toxicological evaluations conducted using human embryo hepatocytes indicated that these products had much lower toxicity than the parental compound, AFB_1_.

The aqueous extract of hyssop, *Micromeria graeca*, was shown to halt AFB_1_ production in *Aspergillus flavus*. The observed inhibitory effect was attributed to the downregulation of specific transcripts within the AF biosynthesis pathway [15]. The proposed approach falls well into green farming practices aiming at reducing the use of fungicides. Similarly, the ability of curcumin to prevent AFB_1_ hepatoxicity was reported. The alleviation in the typical symptoms associated with AFB_1_-induced hepatotoxicity due to curcumin inclusion/supplementation was attributed mainly to the pivotal inhibition of CYP450 isozyme-mediated activation of AFB_1_ to toxic AFBO [16].

The detailed *in silico* analysis of a laccase (and two different isoforms) capable of degrading AFB_1_ and AFM_1_ was reported in this special issue [17]. This interesting investigation helped in pinpointing the structural differences among the three studied isoforms and highlighting the most suitable isoform for future protein engineering approaches.

An exciting report about using *Bacillus subtilis* ANSB060 to ameliorate the negative effects of AFs in ducks is presented [18]. The bacterium was originally isolated from fish gut and showed the ability to protect the growth performance of Cherry Valley ducks fed with moldy maize naturally contaminated with AFs. In a parallel fashion, the ability of *Bacillus licheniformis* CK1 to protect post-weaning gilts from ZEA-contaminated feed was demonstrated. The capability of this bacterium to degrade ZEA was associated with the reported protection mechanism [19]. Finally, the ability of sporoderm-broken spores of *Ganoderma lucidum* to enhance the immune function and maintain the growth performance of broiler chickens exposed to AFB_1_ was detailed. The results showed that diets contaminated with a low level of AFB_1_ can be consumed without any negative consequences as long as they are supplemented with the sporoderm-broken spores of *G. lucidum* [20]. Moreover, the introduced treatment was able to restore the normal levels of IgA and IgG in the serum of chickens exposed to AFB_1_.

The enzymatic modifications of DON occupied a considerable part of this issue, particularly the C3 chemical group modifications. First, Tian et al. suggested that the glycosylation of this group is part of the self-protection mechanism(s) possessed by multiple *Trichoderma* strains serving as antagonists towards *Fusarium graminearum* growth [21]. Similarly, Hassan et al. [22] explored the epimerization of the above group (C3) and its influence on the molecular interactions of DON and its C3 stereoisomer (3-*epi*-DON) with well-defined enzymes such as Tri101 acetyltransferases to conclude that the associated changes within the involved –OH group not only influence DON’s toxicity but also increase the overall polarity of this toxin as well as changing its acetylation patterns [22].

This issue also encompasses some excellent in-depth reviews. The review shared by Loi et al. covered mycotoxins biotransformation by native and commercial enzymes [23], covering purified enzymes isolated from bacteria, fungi, and plants with validated potentialities using *in vitro* and *in vivo* methods and setting examples for applications in food, feed, biogas, and biofuel industries. Zhu et al. brought attention to the most recent strategies and methodologies for developing microbial detoxification systems to mitigate mycotoxins [24], highlighting the tremendous and unexpected challenges facing any progress in this regard including the isolation of single colonies harboring the reported biotransformation activity and the assessment of the cellular toxicity of final biotransformation by-products. The review prepared by Hojnik et al. was dedicated to explore the use of cold atmospheric pressure plasma to decontaminate mycotoxins [25], presenting the advantages of this approach (cost efficiency, ecologically-friendly, negligible influence on food quality and attributes) which may overcome many weaknesses associated with the conventional/classical methods of inactivation. Finally, the mitigation of PAT in fresh and processed food commodities including beverages was discussed by Ioi et al. in a separate review [26] that covered the pre-processing stage (storage conditions, use of fungicides, and the physical removal of fungi and infected tissues). The review further detailed the effects of common processing techniques (including pasteurization, filtration, and fermentation) on PAT and reviewed non-thermal methods (such as high hydrostatic pressure, UV radiation, enzymatic degradations, and binding to microorganisms) to remove or detoxify PAT.

Overall, we are thrilled to present the above genuine contributions with the diligent work of many involved teams that collectively aimed at addressing some of the main challenges that remain within the mycotoxin-mitigation arena using both integrative and innovative approaches. The promising outcomes of this research focus create a foundation to use recombinant enzymes/proteins for the detoxification of many agriculturally-important mycotoxins, including AFs, PAT, and DON. Moreover, the use of innovative processing techniques (such as infrared roasting, non-ionizing radiations, cold atmospheric pressure plasma, and neutral electrolyzed water) will greatly enhance the safety of numerous food/feed commodities with diverse physical attributes/chemical compositions.
